# Quercetin (Que) Improved the Biological Activity of Preeclampsia Cells by Inhibiting Autophagy

**DOI:** 10.1002/fsn3.72166

**Published:** 2026-07-25

**Authors:** Mao Yifan, Ding Shuyun, Xu Rui, Li Yuan, Jiang Feiyun

**Affiliations:** ^1^ Gynecology of the Second People's Hospital of Wuhu City Wuhu City Anhui Province China; ^2^ Department of Geriatrics The First Affiliated Hospital of Wannan Medical College Wuhu City Anhui Province China

**Keywords:** AKT, PE, PI3K, que, THR‐8/SVnoe

## Abstract

Quercetin, a phytochemical sourced from botanicals, exhibits therapeutic potential for conditions including preeclampsia (PE); nevertheless, its precise mechanisms of action against this condition remain incompletely characterized. This investigation sought to explore the hypothesis that quercetin enhances cellular function in PE by suppressing autophagy during in vitro experiments. Within an oxygen‐deprived setting, placental trophoblast cells were generated and subsequently exposed to varying doses of Quercetin (Que) alongside LY294002, a compound inhibiting PI3K activity. Cellular multiplication, programmed cell death, and invasive capacity were quantified. This evaluation employed tetrazolium salt (MTT) testing, fluorescence‐activated cell sorting, Transwell chamber analysis, and scratch wound closure measurements. Furthermore, subcellular architecture was visualized through electron microscopic imaging, while immunoblot analysis determined expression levels of associated proteins. Relative to controls, HTR‐8/SVneo cells exhibited markedly suppressed proliferation, invasive capacity, and 24‐h/48‐h scratch assay closure rates, alongside substantially elevated apoptotic activity (*p* < 0.001 throughout). Concomitantly, protein expression of PI3K, AKT, and mTOR was profoundly diminished, while autophagic activity was significantly amplified (*p* < 0.001 throughout). Subsequent Que intervention significantly enhanced cellular proliferation, invasion, and migratory potential in HTR‐8/SVneo cultures, coupled with attenuated autophagy levels attributable to PI3K/AKT pathway activation. However, co‐administration of Que with LY294002 substantially abrogated these Que‐mediated effects. In conclusion, laboratory investigation demonstrates that Quercetin elevated the functional capabilities of placental trophoblast cells by modulating the phosphatidylinositol 3‐kinase/protein kinase B signaling axis, concurrently suppressing autophagic processes.

## Graphical abstract

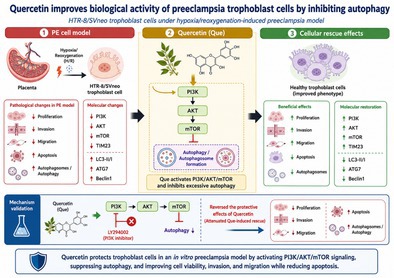

## 1 Introduction

Preeclampsia represents a serious gestational disorder predominantly manifesting beyond the 20‐week mark, characterized by maternal hypertension and potential multi‐organ impairment, notably affecting hepatic and renal systems (Mol et al. 2016). This condition contributes to approximately 76,000 maternal and 500,000 fetal mortalities annually, exhibiting a worldwide prevalence between 2% and 8% (Khan et al. 2006; Tianthong and Phupong 2021). Population studies identify elevated BMI, advanced maternal age, and specific paternal influences (including partner status, inter‐pregnancy intervals, and genetic markers) as significant risk determinants (Giannubilo et al. 2024; Gesuita et al. 2019). Pathological investigations link PE development to dysregulated oxidative pathways and inflammatory responses, potentially driven by xanthine oxidase activity and STAT3 signaling anomalies (Annesi et al. 2024; Marzioni et al. 2025). Additionally, enhanced autophagic processes in placental trophoblasts demonstrate a critical association with preeclampsia pathogenesis (Li et al. 2021).

Quercetin (Que), a naturally occurring flavonoid compound derived from diverse plant sources including fruits and vegetables, exhibits anti‐inflammatory, antioxidant, and vasodilatory properties, and contributes to the prevention and management of cardiovascular, cerebrovascular, and neurodegenerative disorders (Ezzati et al. 2020; Alshehri et al. 2022; Amanzadeh et al. 2019). Research indicates Que counteracts oxidative damage resulting from vascular endothelial impairment in preeclampsia (PE) rat models through activation of the Nrf2/ARE pathway (Zhang et al. 2025). However, the potential influence of Que on enhancing placental trophoblast cellular functionality via autophagy suppression remains unexplored. Consequently, this investigation developed an in vitro PE model using hypoxic conditions, administering varied Que concentrations for evaluation. Furthermore, the therapeutic mechanism of Que in PE was examined through targeted inhibition of the PI3K signaling cascade.

## 2 Materials and Methods

### 2.1 Cell Line

The human trophoblast HTR‐8/Svneo cell linewas purchased from Wuhan Punosai Life Technology Co. Ltd. The HTR‐8/Svneo cells were maintained in RPMI1640 medium containing 10% fetal bovine serum and 1% penicillin streptomycin, and cultured in an environment of 37°C and 5% CO_2_.

### 2.2 Drugs and Main Reagents

Que was purchased from Sigma‐Aldrich (Merck KGaA; purity, ≥ 95%); the MTT assay kit was purchased from Nanjing Kaiji Biotechnology Co. Ltd.; the RIPA solution was purchased from Shanghai Biyuntian Biotechnology Co. Ltd.; rabbit‐derived primary antibodies, PI3K, AKT, mTOR, Light Chain 3 (LC3), ATG7, Beclin1, TIM23, and GAPDH were purchased from Abcam; and LY294002 (PI3K inhibitor) was purchased from Sigma‐Aldrich (Merck KGaA).

### 2.3 Main Instruments

The following main instruments were used: Ultra‐thin slicer (Leica EM UC7; Leica Microsystems GmbH); multi‐functional enzyme‐linked immunosorbent assay (iMark680; Bio Rad Laboratories Inc.); inverted fluorescence microscope (IX73; Olympus Corp.); and transmission electron microscope (JEM‐1230; JEOL Ltd.).

### 2.4 Construction of PE Cell Model and Cell Grouping

The PE cell model was established by inducing hypoxia reoxygenation for 1 h (H1R1). HTR‐8/Svneo cells were divided into the following groups: Normal, model, Que‐L, Que‐M, Que‐H, Que, and Que + LY294002. Except for the normal group, all other groups contained constructed hypoxic PE cell models. For the Que‐L, Que‐M, and Que‐H groups, HTR‐8/Svneo cells were treated with 0.5, 1.0, or 2.0 mg/L Que, respectively, and then PE was induced using a hypoxic environment; after administering 2.0 mg/L Que intervention to HTR‐8/Svneo cells in the Que group, the PE cell model was constructed using a hypoxic environment (1% O_2_, 5% CO_2_, and 94% N2); to construct the Que + LY294002 group, HTR‐8/Svneo cells were treated with 2.0 mg/L Que and 5 μM LY294002, respectively (Zhang et al. 2025; Liu et al. 2023).

### 2.5 MTT Assay for Detecting Cell Proliferation

HTR‐8/Svneo cells were seeded into 96 well plates at a density of 2 × 10^4^ cells/well, with a cell density of 200 μL per well (1 × 10^5^ cells/mL). After grouping and treatment for 72 h, 40 μL MTT solution (cat. no. 0793; AMRESCO Inc.; 5 mg/mL) was added to each well and incubated for 2 h. The absorbance value of each group of cells was measured at 570 nm to evaluate the rate of cell proliferation (Mosmann 1983).

### 2.6 Observation of Cellular Autophagy Using Transmission Electron Microscopy

Each group of cells was fixed with 2.5% glutaraldehyde‐1% citric acid, washed with PBS, dehydrated with gradient ethanol and acetone, embedded in pure 812 embedding agent and polymerized in a 60°C oven for 48 h. The sample was then cut into ultra‐thin sections (60–80 nm) using an ultra‐thin slicer. Slices were stained with 3% uranium acetate lead citrate double staining, and the number of autophagosomes was observed under a transmission electron microscope (Klionsky et al. 2021).

### 2.7 Wound Healing to Assess Cell Migration

HTR‐8/Svneo cells in the logarithmic growth phase were inoculated into a 60‐mm culture dish at a density of 1 × 10^5^ cells/mL, and cultured for 24 h. A scratch was made using the tip of a sterile blue pipette, and the cells were subsequently grouped and cultured for 48 h. Images were captured under an inverted microscope at the same position at 0, 24 and 48 h to calculate the wound healing rate (Liang et al. 2007).

### 2.8 Transwell Assay for Evaluating the Rate of Cell Invasion

After grouping the HTR‐8/Svneo cells, they were resuspended in serum‐free culture medium (2 × 10^4^ cells/mL). Subsequently, 200 μL cell suspension was added to the upper Transwell chamber (pre‐coated with Matrigel) and 600 μL culture medium (containing 10% fetal bovine serum) was added to the lower chamber. After 48 h of incubation, the transmembrane cells were fixed in 4% paraformaldehyde for 30 min and stained using crystal violet staining solution for 20 min. The number of invading cells was counted under an inverted microscope (Justus et al. 2014).

### 2.9 Detection of Cell Apoptosis Using Flow Cytometry

After grouping HTR‐8/Svneo cells, they were resuspended in serum‐free culture medium (2 × 10^4^ cells/mL), washed twice with pre‐cooled PBS and incubated at room temperature with 100 μL 1X Annexin V working solution containing PI (1 mg/L) in the dark for 15 min. Subsequently, 400 μL 1X binding buffer was added and rapidly vortexed, and the proportion of late‐stage apoptotic cells was immediately analyzed using flow cytometry (Vermes et al. 1995).

### 2.10 Western Blot Detection of Related Protein Expression

The total protein of HRT‐8/Svneo cells in each group was extracted using RIPA lysis solution after the treatment of each group. After measuring the protein concentration, sodium dodecyl sulfate‐polyacrylamide gel electrophoresis was used to separate the protein samples (30 μg), which were then transferred to a polyvinylidene fluoride membrane. The membrane was fixed using 5% skim milk for 1 h and incubated overnight with the following primary antibodies: PI3K (cat. no. ab302958; Abcam), AKT (cat. no. ab8933; Abcam), mTOR (cat. no. ab313429; Abcam), LC3 (cat. no. ab192890; Abcam), ATG7 (cat. no. ab52472; Abcam), Beclin1 (cat. no. ab302669; Abcam), TIM23 (cat. no. ab230253; Abcam) and GAPDH (cat. no. ab8245; Abcam) (all 1:1000) in the closed solution at 4°C. The membrane was then incubated with secondary antibodies (1:4000; cat. no. KGC6202‐0.1; cat. no. KGC6203‐0.1; Nanjing KeyGenBiotech Co. Ltd.) (1:4000; cat. no. ab6759; Abcam) at room temperature for 1 h. Finally, it was developed, the grayscale value of the bands was detected and the expression level of the target protein was calculated (Towbin et al. 1979).

### 2.11 Statistical Analysis

Employing SPSS 22.0 software (IBM Corp.), we expressed all continuous measurements as the mean ± standard deviation. The data were first tested for conformity to parametric assumptions: normality with the Kolmogorov–Smirnov test and variance homogeneity with Levene's test. For comparing multiple groups, a one‐way analysis of variance (ANOVA) was employed. In cases where the ANOVA indicated significance, Tukey's honest significant difference (HSD) test was utilized for post hoc comparisons. The threshold for statistical significance was set at a *p*‐value of 0.05 (Kim 2017).

## 3 Results

### 3.1 Que Increased Rate of Cell Proliferation and Apoptosis

**FIGURE 1:**
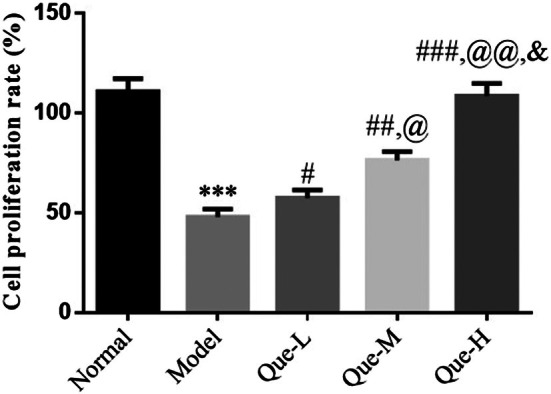
Que's treatment effects to cell proliferation by MTT assay. Normal: HTR/Svneo cells were treated with normal; Model: HTR/Svneo cells were treated with hypoxic environment; Que‐L: HTR/Svneo cells were treated with 0.5 mg/L Que in hypoxic environment; Que‐M: HTR/Svneo cells were treated with 1.0 mg/L Que in hypoxic environment; Que‐H: HTR/Svneo cells were treated with 2.0 mg/L Que in hypoxic environment (*n* = 3). ***p < 0.001, compared with normal group; #*p* < 0.05, ##*p* < 0.01, ###*p* < 0.001, compared with model group; @*p* < 0.05, @@*p* < 0.01, compared with Que‐L group; &*p* < 0.05, compared with Que‐M group.

**FIGURE 2:**
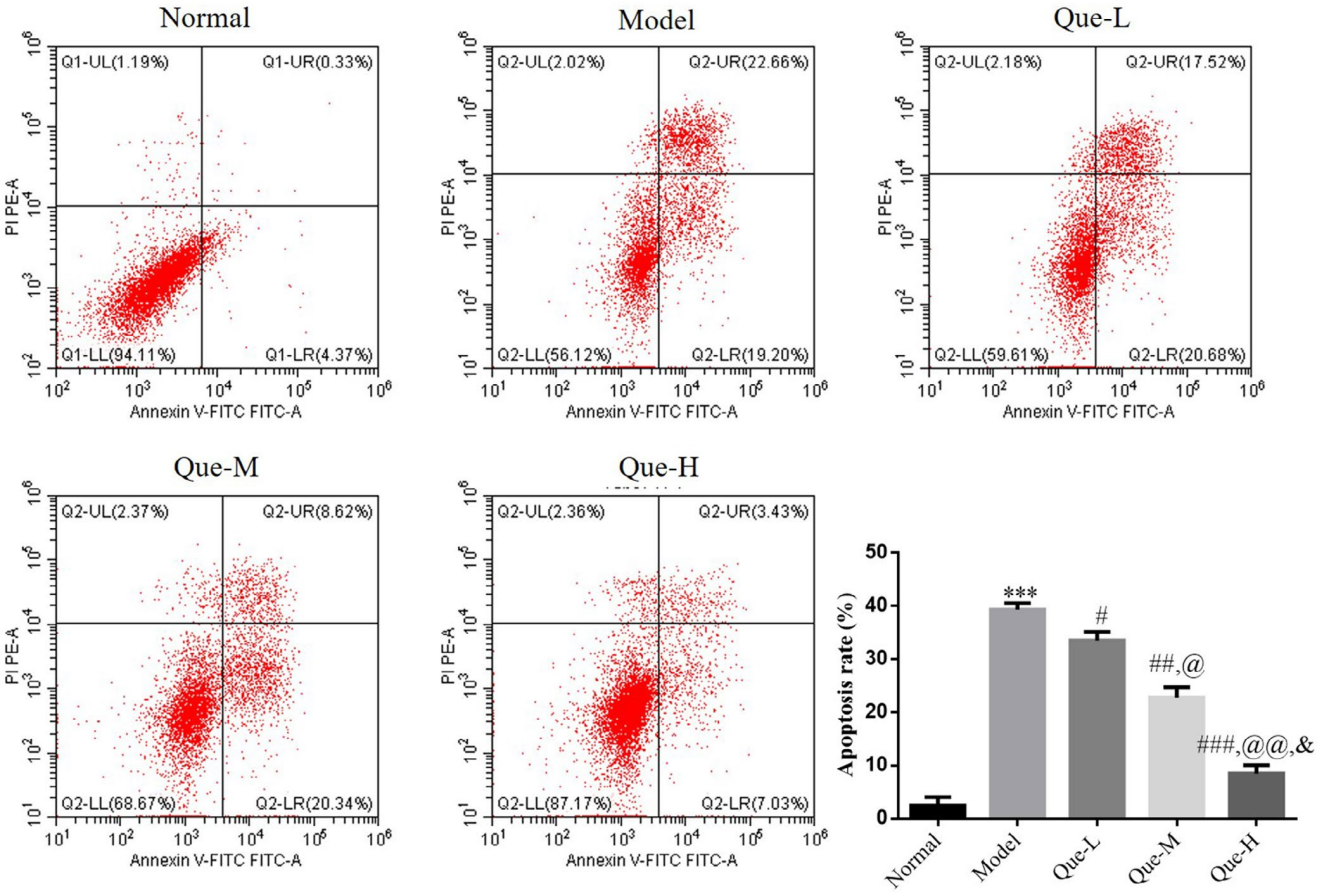
Que's treatment effects on cell apoptosis by flow cytometry. Normal: HTR/Svneo cells were treated with normal; Model: HTR/Svneo cells were treated with a hypoxic environment; Que‐L: HTR/Svneo cells were treated with 0.5 mg/L Que in a hypoxic environment; Que‐M: HTR/Svneo cells were treated with 1.0 mg/L Que in a hypoxic environment; Que‐H: HTR/Svneo cells were treated with 2.0 mg/L Que in a hypoxic environment (*n* = 3). ***p < 0.001, compared with Normal group; #*p* < 0.05, ##*p* < 0.01, ###*p* < 0.001, compared with Model group; @*p* < 0.05, @@*p* < 0.01, compared with Que‐L group; &*p* < 0.05, compared with Que‐M group.

TheMTT assay demonstrated that cell proliferation significantly decreased (*p* < 0.001; Figure 1) and cell apoptosis significantly increased (*p* < 0.001; Figure 2) in the model group. After Que treatment, cell proliferation was significantly increased and cell apoptosis was significantly decreased in the Que‐treated groups (Que‐L, Que‐M and Que‐H groups), compared with the model group (*p* < 0.05, respectively; Figures 1 and 2). Moreover, there were significant differences in cell proliferation and cell apoptosis rates among the Que‐treated groups (*p* < 0.05, respectively; Figures 1 and 2).

### 3.2 Que Enhances Cell Invasion and Migration

**FIGURE 3:**
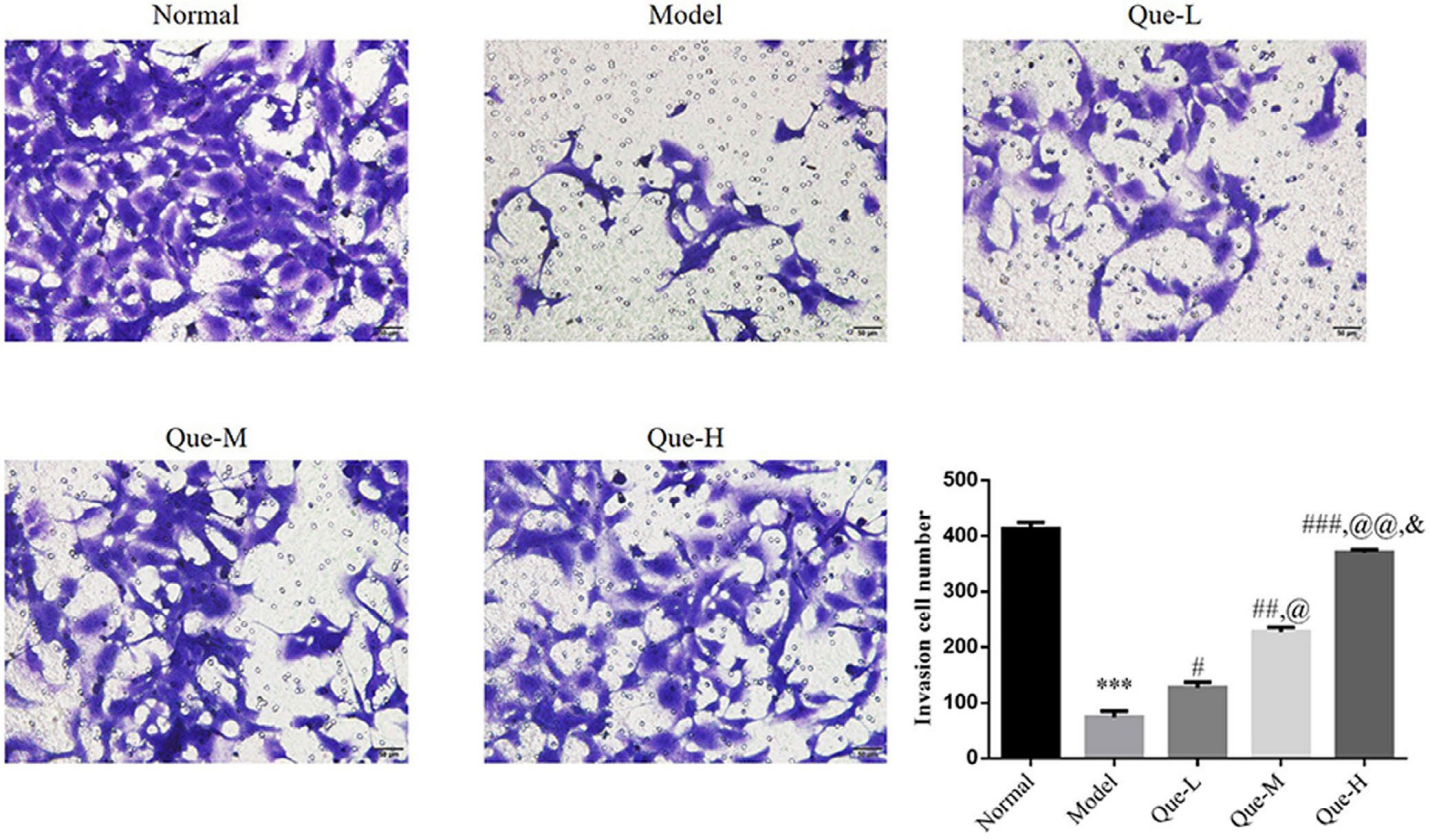
Que's treatment effects to invasion cell number by transwell assay (200×). Normal: HTR/Svneo cells were treated with normal; Model: HTR/Svneo cells were treated with hypoxic environment; Que‐L: HTR/Svneo cells were treated with 0.5 mg/L Que in hypoxic environment; Que‐M: HTR/Svneo cells were treated with 1.0 mg/L Que in hypoxic environment; Que‐H: HTR/Svneo cells were treated with 2.0 mg/L Que in hypoxic environment (*n* = 3). ***p < 0.001, compared with Normal group; #*p* < 0.05, ##*p* < 0.01, ###*p* < 0.001, compared with Model group; @*p* < 0.05, @@*p* < 0.01, compared with Que‐L group; &*p* < 0.05, compared with Que‐M group.

**FIGURE 4:**
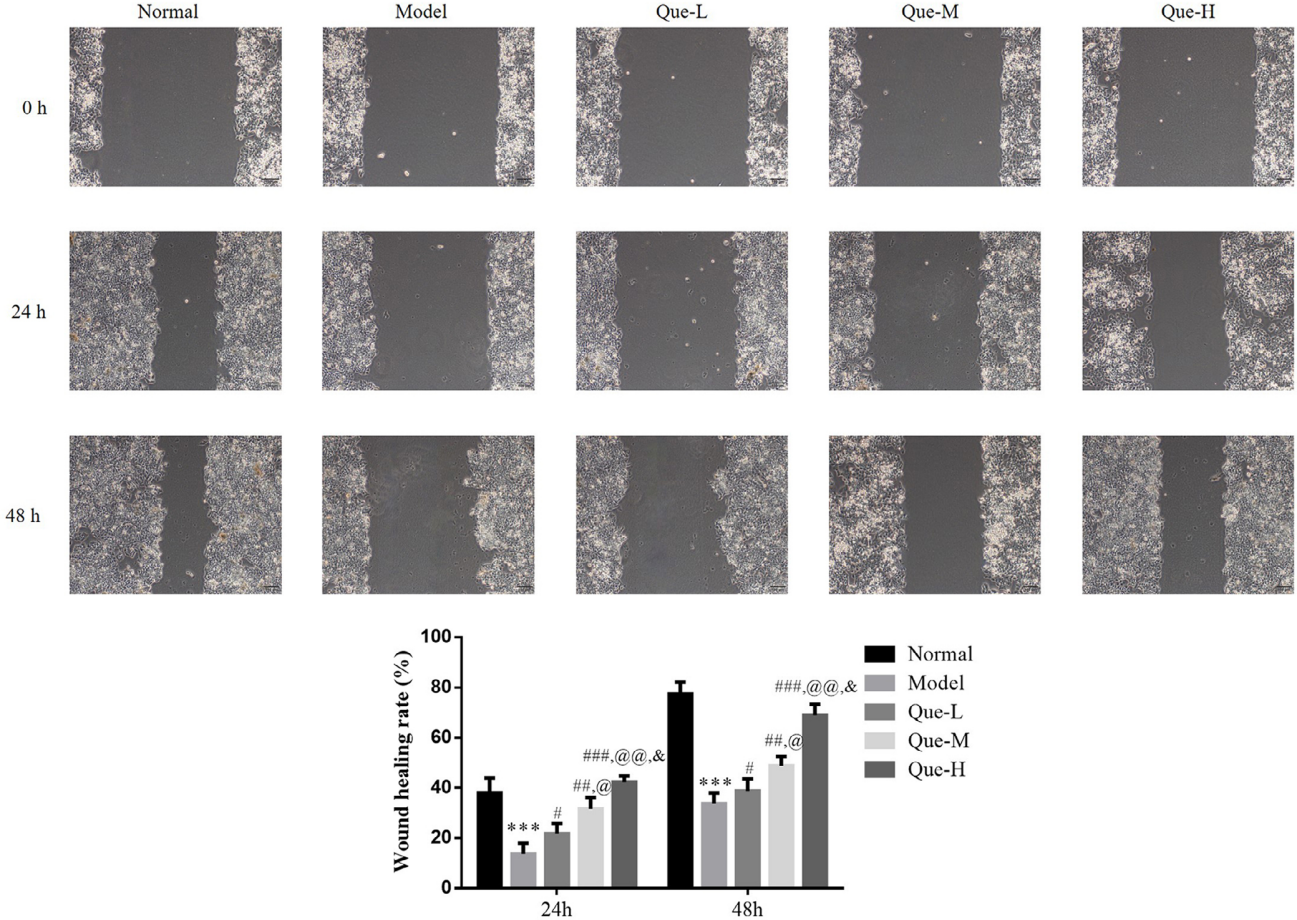
Que's treatment effects to 24 h and 48 h wound healing rates by wound healing assay (100×) (*n* = 3). Normal: HTR/Svneo cells were treated with normal; Model: HTR/Svneo cells were treated with hypoxic environment; Que‐L: HTR/Svneo cells were treated with 0.5 mg/L Que in hypoxic environment; Que‐M: HTR/Svneo cells were treated with 1.0 mg/L Que in hypoxic environment; Que‐H: HTR/Svneo cells were treated with 2.0 mg/L Que in hypoxic environment. ***p < 0.001, compared with Normal group; #*p* < 0.05, ##*p* < 0.01, ###*p* < 0.001, compared with Model group; @*p* < 0.05, @@*p* < 0.01, compared with Que‐L group; &*p* < 0.05, compared with Que‐M group.

Assessment of cellular metastatic properties revealed that the model group had severely compromised abilities in both invasion (Transwell assay) and migration (wound healing at 24/48 h) compared to normal controls (*p* < 0.001 for all; Figures 3 and 4). This deficit was effectively mitigated by Que treatment, with all dosage groups showing significant recovery of these functions versus the model group (*p* < 0.05). Importantly, the efficacy of Que exhibited a dose‐responsive manner, as significant differences in both invasion and migration metrics were observed among the Que‐L, Que‐M, and Que‐H groups (*p* < 0.05; Figures 3 and 4).

### 3.3 Que Treatment Affects Cell Autophagy

**FIGURE 5:**
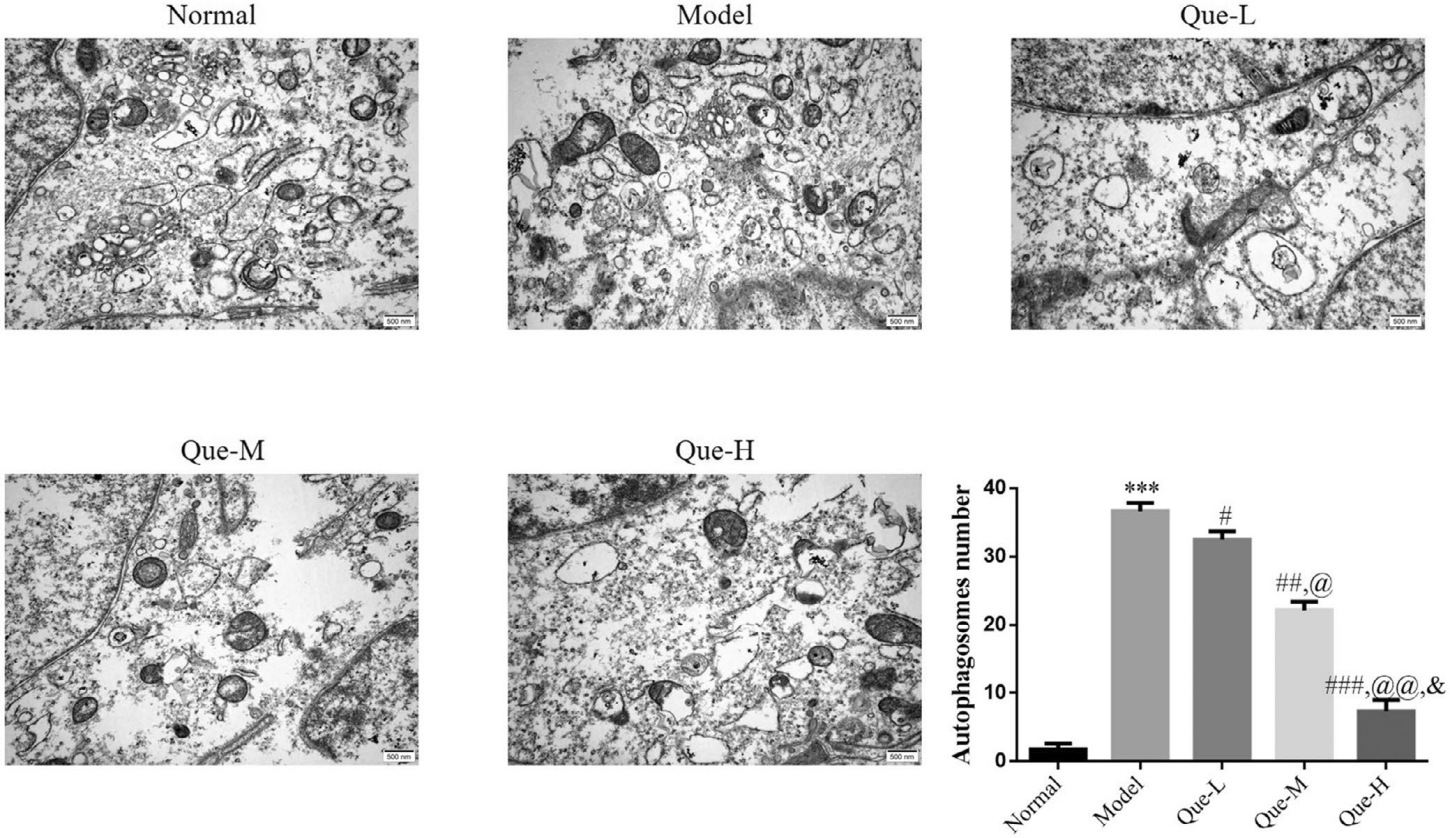
Que's treatment effects to autophagosome by TEM (20,000×). Normal: HTR/Svneo cell were treated with normal; Model: HTR/Svneo cell were treated with hypoxic environment; Que‐L: HTR/Svneo cell were treated 0.5 mg/L Que in hypoxic environment; Que‐M: HTR/Svneo cell were treated 1.0 mg/L Que in hypoxic environment; Que‐H: HTR/Svneo cell were treated 2.0 mg/L Que in hypoxic environment (*n* = 3). ***p < 0.001, *compared with n*ormal group; #*p* < 0.05, ##*p* < 0.01, ###*p* < 0.001, compared with model group; @*p* < 0.05, @@*p* < 0.01, compared with Que‐L group; &*p* < 0.05, compared with Que‐M group.

TEM demonstrated that there were no autophagosomes in the normal group, and compared with this group, the autophagosome number was significantly increased in the model group (*p* < 0.001; Figure 5). Moreover, compared with the model group, the autophagosome number significantly decreased in the Que‐L, Que‐M, and Que‐H groups, with dose‐dependent decreases observed (*p* < 0.05, respectively; Figure 5).

### 3.4 Que Affects the Relative Protein Expression of PI3K, AKT, mTOR, LC3, ATG7, Beclin1 and TIM23

**FIGURE 6:**
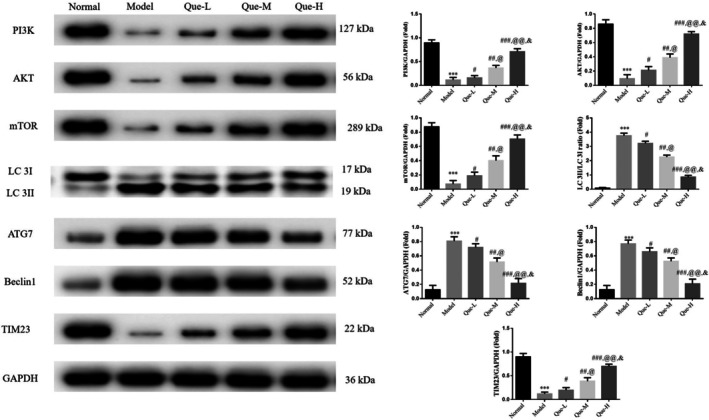
Que's treatment effects to relative protein expression by WB assay. Normal: HTR/Svneo cells were treated with normal; Model: HTR/Svneo cells were treated with hypoxic environment; Que‐L: HTR/Svneo cells were treated with 0.5 mg/L Que in hypoxic environment; Que‐M: HTR/Svneo cells were treated with 1.0 mg/L Que in hypoxic environment; Que‐H: HTR/Svneo cells were treated with 2.0 mg/L Que in hypoxic environment (*n* = 3). ***p < 0.001, compared with normal group; #*p* < 0.05, ##*p* < 0.01, ###*p* < 0.001, compared with model group; @*p* < 0.05, @@*p* < 0.01, compared with Que‐L group; &*p* < 0.05, compared with Que‐M group.

Western blotting revealed that PI3K, AKT, mTOR, and TIM23 protein levels were significantly decreased, and the LC3II/LC3I ratio, ATG7, and Beclin1 protein levels were significantly increased in the model group, compared with the normal group (*p* < 0.001, respectively; Figure 6). Furthermore, following Que supplementation, PI3K, AKT, mTOR, and TIM23 protein levels were significantly increased, and the LC3II/LC3I ratio, ATG7, and Beclin1 protein levels were significantly decreased in the Que‐L, Que‐M, and Que‐H groups, compared with the model group, with dose‐dependent changes observed (*p* < 0.05, respectively; Figure 6).

### 3.5 Impact of LY294002 Treatment on Cell Proliferation and Apoptosis After Treatment With Que

**FIGURE 7:**
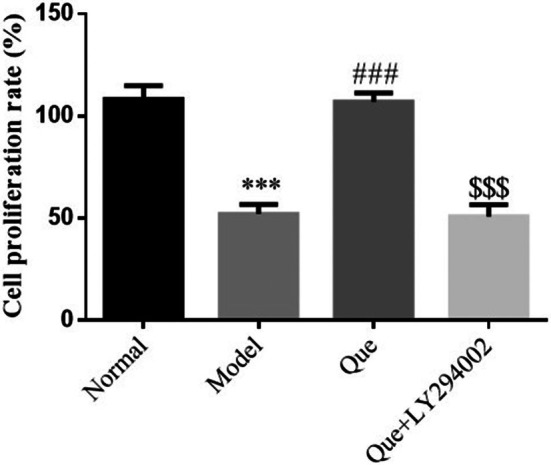
LY294002's effects to Que's treatment effect in cell proliferation by MTT assay. Normal: HTR/Svneo cell were treated with normal; Model: HTR/Svneo cell were treated with hypoxic environment; Que: HTR/Svneo cell were treated 2.0 mg/L Que in hypoxic environment; Que + LY294002: HTR/Svneo cell were treated 2.0 mg/L Que and 5 μ M LY294002 in hypoxic environment (*n* = 3). ***p < 0.001, compared with Normal group; ###*p* < 0.001, compared with Model group; $$$*p* < 0.001, compared with Que group.

**FIGURE 8:**
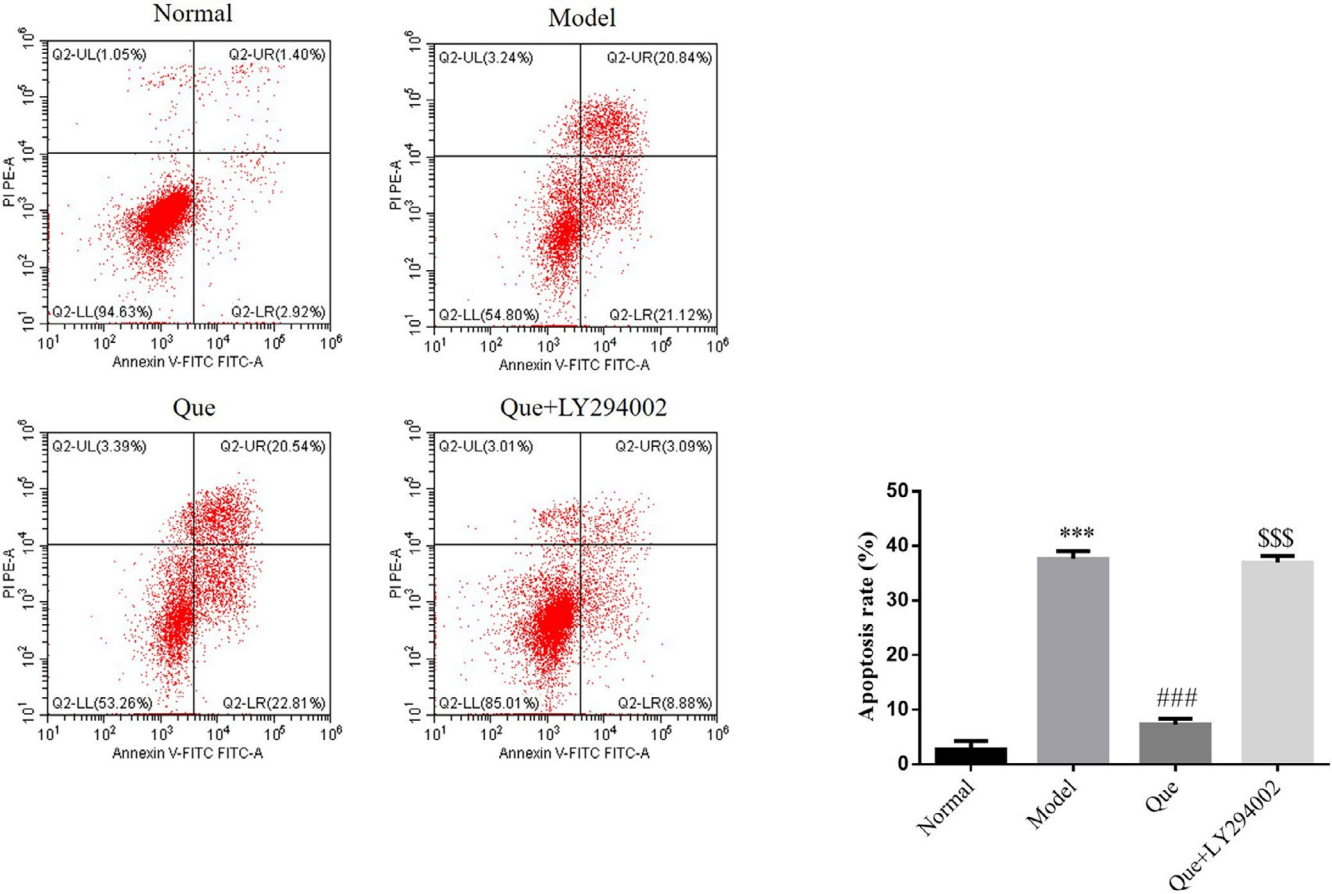
LY294002's effects to Que's treatment effect in cell apoptosis rates by flow cytometry (*n* = 3). Normal: HTR/Svneo cells were treated with normal; Model: HTR/Svneo cells were treated with a hypoxic environment; Que: HTR/Svneo cells were treated with 2.0 mg/L Que in a hypoxic environment; Que + LY294002: HTR/Svneo cells were treated with 2.0 mg/L Que and 5 μM LY294002 in a hypoxic environment. ***p < 0.001, compared with normal group; ###*p* < 0.001, compared with model group; $$$*p* < 0.001, compared with Que group.

The MTT assay demonstrated that cell proliferation was significantly decreased (*p* < 0.001; Figure 7) and the rate of cell apoptosis was significantly increased (*p* < 0.001; Figure 8) in the model group, compared with the normal group. Following treatment with Que, the rate of cell proliferation was significantly increased and the rate of cell apoptosis was significantly decreased in the Que‐treated groups, compared with the model group (*p* < 0.001, respectively; Figures 7 and 8). However, with Que + LY294002 treatment, the rate of cell proliferation was significantly decreased and the rate of cell apoptosis was significantly increased in the Que + LY294002 group, compared with the Que‐treated groups (*p* < 0.001, respectively; Figures 7 and 8).

### 3.6 Effects of LY294002 Onthe Rate of Invasion and Migration Following Que Treatment

**FIGURE 9:**
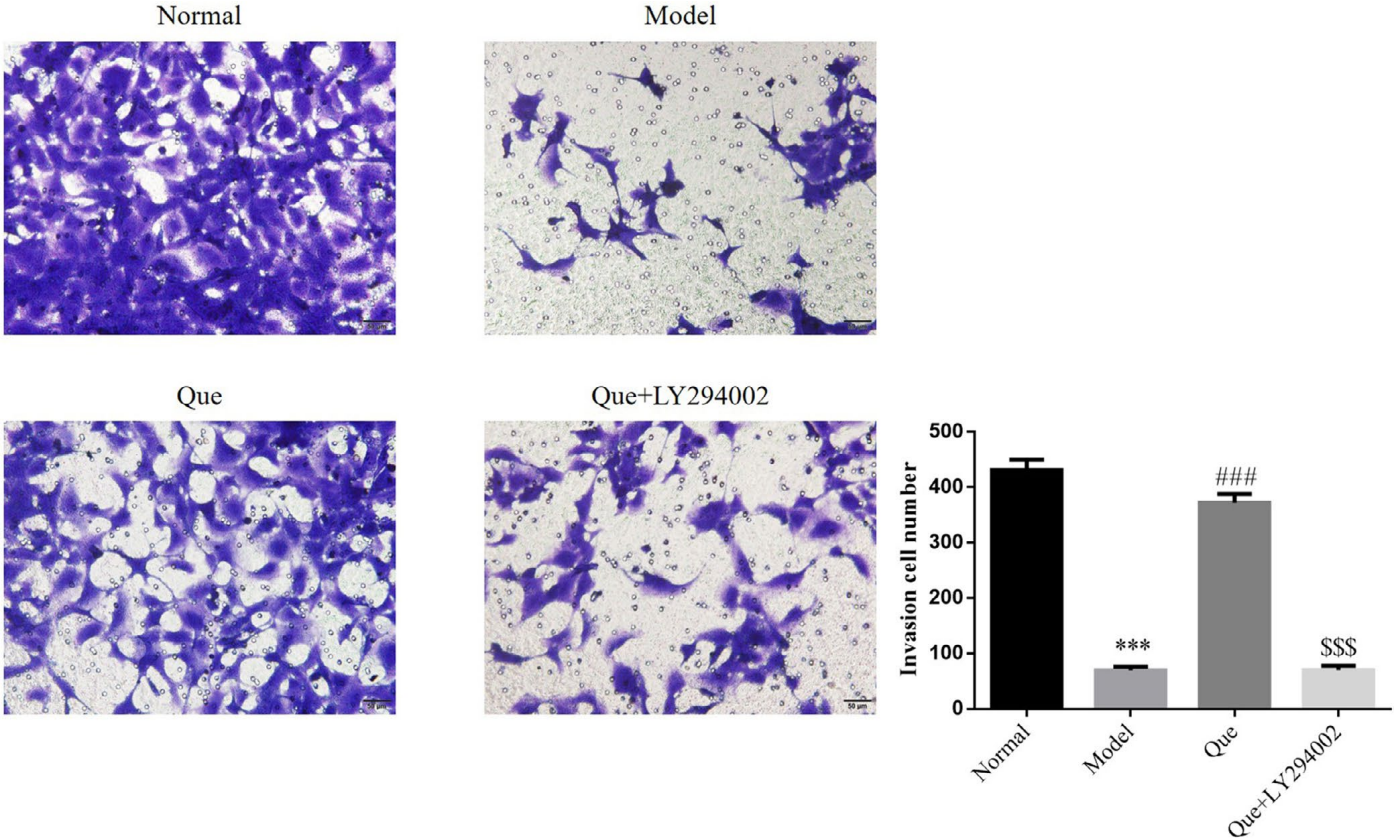
LY294002's effects to Que's treatment effects to invasion cell number by transwell assay (200×) (*n* = 3). Normal: HTR/Svneo cells were treated with normal; Model: HTR/Svneo cells were treated with hypoxic environment; Que: HTR/Svneo cells were treated 2.0 mg/L Que in hypoxic environment; Que + LY294002: HTR/Svneo cells were treated 2.0 mg/L Que and 5 μM LY294002 in hypoxic environment. ***p < 0.001, compared with Normal group; ###*p* < 0.001, compared with Model group; $$$*p* < 0.001, compared with Que group.

**FIGURE 10:**
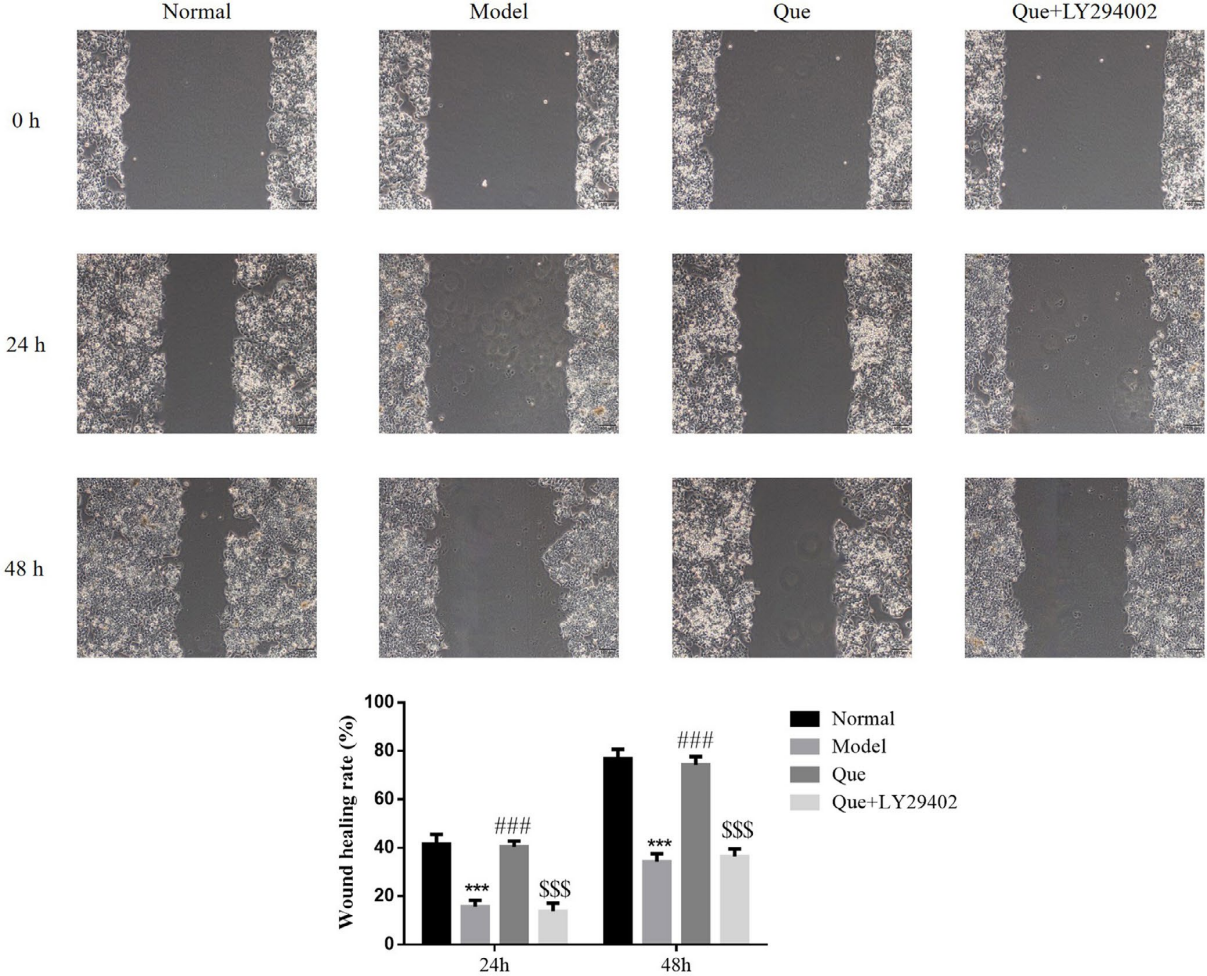
LY294002's effects to Que's treatment effects to 24 h and 48 h wound healing rates by wound healing assay (100×) (*n* = 3). Normal: HTR/Svneo cells were treated with normal; Model: HTR/Svneo cells were treated with hypoxic environment; Que: HTR/Svneo cells were treated 2.0 mg/L Que in hypoxic environment; Que + LY294002: HTR/Svneo cells were treated 2.0 mg/L Que and 5 μM LY294002 in hypoxic environment. ***p < 0.001, compared with Normal group; ###*p* < 0.001, compared with Model group; $$$*p* < 0.001, compared with Que group.

Transwell and wound healing assays demonstrated that the invasion cell number and 24‐ and 48‐h wound healing rates were significantly decreased in the model group, compared with the normal group (*p* < 0.001, respectively; Figures 9 and 10). Furthermore, following Que treatment, the invasion cell number and 24‐ and 48‐h wound healing rates were significantly increased in the Que‐treated groups, compared with the model group (*p* < 0.05, respectively; Figures 9 and 10). However, after treatment with Que + LY294002, the invasion cell number and 24‐ and 48‐h wound healing rates were significantly decreased in the Que + LY294002 group, compared with the Que‐treated groups (*p* < 0.001, respectively; Figures 9 and 10).

### 3.7 Effects of LY294002 Treatment on the Number of Autophagosomes Following Treatment With Que

**FIGURE 11:**
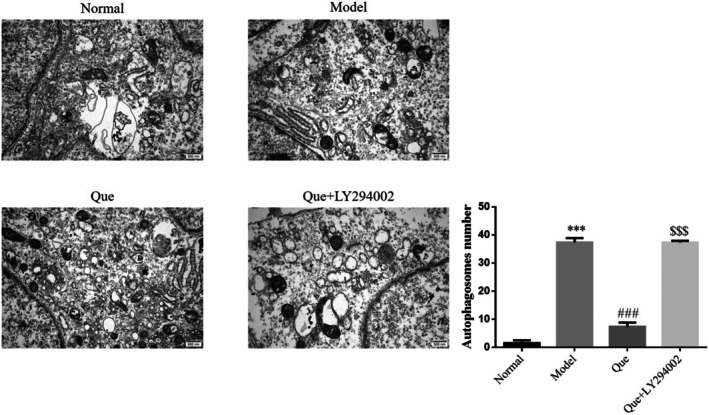
LY294002's effects to Que's treatment effects to autophagosome by TEM (20,000×) (*n* = 3). Normal: HTR/Svneo cells were treated with normal; Model: HTR/Svneo cells were treated with hypoxic environment; Que: HTR/Svneo cells were treated 2.0 mg/L Que in hypoxic environment; Que + LY294002: HTR/Svneo cells were treated 2.0 mg/L Que and 5 μM LY294002 in hypoxic environment. ***p < 0.001, compared with normal group; ###*p* < 0.001, compared with model group; $$$*p* < 0.001, compared with Que group.

TEM revealed that there were no autophagosomes in the normal group, and compared with this group, the autophagosome number was significantly increased in the model group (*p* < 0.001; Figure 11). Moreover, compared with the model group, the autophagosome number was significantly decreased in the Que‐treated groups (*p* < 0.001, respectively; Figure 11); however, following Que + LY294002 supplementation, the autophagosome number was significantly increased in the Que + LY294002 group compared with the Que‐treated groups (*p* < 0.001; Figure 11).

### 3.8 Effects of LY294002 Treatment on Relative Proteins Levels of PI3K, AKT, mTOR, LC3, ATG7, Beclin1 and TIM23 After Que Treatment

**FIGURE 12:**
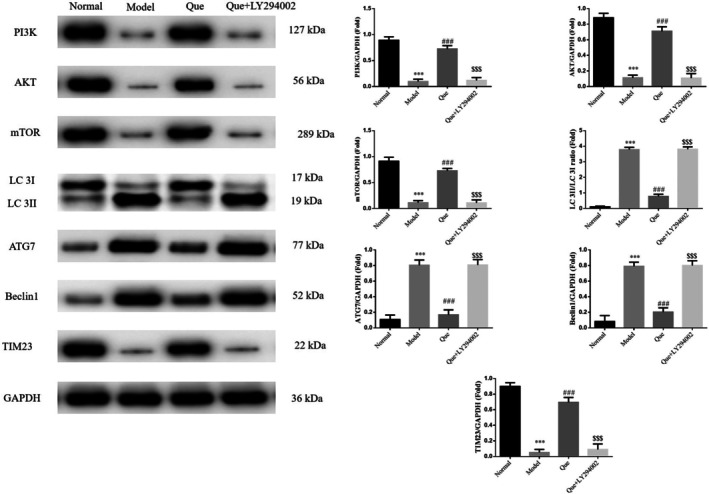
LY294002's effects to Que's treatment effects to relative proteins expression by WB assay (*n* = 3). Normal: HTR/Svneo cell were treated with normal; Model: HTR/Svneo cell were treated with hypoxic environment; Que: HTR/Svneo cell were treated 2.0 mg/L Que in hypoxic environment; Que + LY294002: HTR/Svneo cell were treated 2.0 mg/L Que and 5 μ M LY294002 in hypoxic environment. ***p < 0.001, compared with Normal group; ###*p* < 0.001, compared with Model group; $$$*p* < 0.001, compared with Que group.

Western blotting revealed that, compared with the normal group, PI3K, AKT, mTOR, and TIM23 protein levels were significantly decreased, and the LC3II/LC3I ratio, ATG7, and Beclin1 proteins levels were significantly increased in the model group (*p* < 0.001, respectively; Figure 12). Moreover, after Que supplementation, PI3K, AKT, mTOR, and TIM23 protein levels were significantly increased, and the LC3II/LC3I ratio, ATG7, and Beclin1 proteins levels were significantly decreased in the Que‐treated groups, compared with the model group (*p* < 0.001, respectively; Figure 12). However, following treatment with Que + LY294002, PI3K, AKT, mTOR, and TIM23 protein levels were significantly decreased, and the LC3II/LC3I ratio, ATG7, and Beclin1 proteins levels were significantly increased in the Que + LY294002 group, compared with the Que‐treated groups (*p* < 0.001, respectively; Figure 12).

## 4 Discussion

During uncomplicated gestation, placental trophoblasts infiltrate the uterine lining, transforming high‐resistance spiral arterioles into a low‐impedance circulatory network; however, this vascular adaptation is impaired in preeclampsia (PE), potentially arising from altered maternal‐fetal immunological interactions that may induce placental hypoperfusion (Zhou et al. 2022). PE has historically been characterized as a placental ischemic disorder, wherein oxygen deprivation compromises cellular migratory capacity and hinders trophoblast differentiation toward invasive phenotypes (Li et al. 2024). Furthermore, studies indicate hypoxic conditions activate the PI3K pathway within trophoblasts, effectively establishing a PE simulation model using HTR8/Svneo cells (Selvakumar et al. 2024). Accordingly, this investigation generated a PE trophoblast model via oxygen deprivation. Findings demonstrated that relative to normoxic controls, hypoxia significantly diminished trophoblast migration and invasion capacities while suppressing the PI3K/AKT cascade.

Quercetin exhibits properties including antioxidant, antitumor, and anti‐inflammatory activities (Lin et al. 2024; Chen 2024; Jiang et al. 2024). Nevertheless, to the best of our understanding, existing literature lacks information on quercetin's potential to preserve the functional capacity of HTR‐8/Svneo trophoblast cells through autophagy suppression. In the context of gestation, trophoblasts undergo critical processes enabling proliferation, tissue invasion, and motility, facilitated by a transition akin to epithelial‐mesenchymal change, ultimately permitting endometrial infiltration and vascular remodeling. Impairments in placental development are strongly linked to diminished trophoblast functions—specifically proliferation, invasion, and migration (Kanda et al. 2024). Our investigation revealed that exposure to quercetin at concentrations of 0.5, 1.0, and 2.0 mg/L markedly enhanced the proliferation, invasive potential, and migratory capacity of HTR‐8/Svneo cells subjected to hypoxic conditions. Furthermore, protein analysis via western blotting showed that quercetin administration correlated with elevated levels of PI3K and AKT expression. These collective findings suggest quercetin may augment the biological functionality of hypoxic HTR‐8/Svneo cells. Although quercetin plays a significant role in preeclampsia (PE), the precise molecular pathways governing its regulation of HTR‐8/Svneo cell activity remain undefined.

Autophagy plays a critical functional role during embryonic development, implantation, and gestation. Existing literature indicates dysregulated autophagy mechanisms in preeclampsia (PE), linking this process to placental dysfunction—a hallmark pathological feature observed in PE (Nakashima et al. 2019, 2020). Paradoxically, placental overactivation of autophagy also represents a significant characteristic in PE pathogenesis (Wang et al. 2024; Goudarzi et al. 2024). Trophoblast biological activity diminishes during early gestation following mTOR suppression, which triggers excessive autophagic activation (Knuth et al. 2015; Yang et al. 2020). These observations collectively demonstrate mTOR‐mediated regulation of trophoblast function. As an upstream modulator of mTOR, the PI3K/AKT signaling pathway critically governs cellular autophagy (Goudarzi et al. 2024; Ebrahim et al. 2024). Current experimental data reveal that hypoxic stimulation markedly downregulated PI3K, AKT, and mTOR expression in HTR‐8/Svneo trophoblasts, while concurrently elevating autophagy indicators (LC3II/LC3I ratio, ATG7, Beclin1) (Liang et al. 2024; Chen et al. 2024) and reducing TIM23 protein (Akabane et al. 2023). This evidences heightened autophagy coinciding with diminished cellular function in these cells, aligning with certain investigations (Tsai et al. 2021) yet diverging from others (Nakashima et al. 2019, 2020), potentially reflecting autophagy's dual regulatory nature. We propose that profound PI3K/AKT suppression in PE placentas may induce mTOR inhibition‐driven excessive autophagy. In this study, quercetin (Que) treatment elevated PI3K/AKT expression in HTR‐8/Svneo cells, promoting mTOR activation that subsequently suppressed autophagy and enhanced cellular activity. Critically, PI3K/AKT inhibitor LY294002 substantially counteracted Que's beneficial effects on cellular function. These findings imply Que potentially activates mTOR via PI3K/AKT upregulation, thereby restraining pathological trophoblast autophagy and augmenting biological activity. This positions Que as a prospective protective agent against PE, warranting further investigation through PE murine models and clinical trials. However, there were some limits in our present study; this study just discusses quercetin's treatment effects on PE by vitro study; it has been unclear that quercetin's effects on PE in vivo. In our future study, we will continue this study in vivo.

Collectively, these findings indicate that quercetin (Que) potentially augments trophoblast functionality and ameliorates preeclampsia manifestations, with its primary mechanism appearing centered on modulation of placental PI3K/AKT/mTOR signaling to regulate overactivated autophagic processes. Nevertheless, these experimental observations necessitate additional verification through comprehensive in vivo experimentation before clinical applicability can be established.

## 5 Conclusion

The in vitro experimental results showed that quercetin has the potential to improve PE, and its mechanism may be to exert a protective effect by regulating the PI3K/AKT signaling pathway to inhibit cell autophagy.

## Author Contributions

**Mao Yifan:** visualization, validation. **Jiang Feiyun:** visualization, validation, formal analysis, project administration. **Xu Rui:** validation, formal analysis, project administration. **Ding Shuyun:** validation, software. **Li Yuan:** visualization, methodology, project administration.

## Funding

Anhui Provincial Health Science and Technology Project (AHWJ2025Ab40006) and 2022 Huatuo Talent Program of Wuhu City, Anhui Province.

## Ethics Statement

The authors have nothing to report.

## Consent

The authors have nothing to report.

## Conflicts of Interest

The authors declare no conflicts of interest.

## Data Availability

Data sharing not applicable to this article as no datasets were generated or analyzed during the current study.
